# Life cycle assessment (LCA) of circular consumer electronics based on IC recycling and emerging PCB assembly materials

**DOI:** 10.1038/s41598-024-79732-1

**Published:** 2024-11-25

**Authors:** Tianwei Zhang, Andrew Bainbridge, Jonathon Harwell, Shoushou Zhang, Mahmoud Wagih, Jeff Kettle

**Affiliations:** 1 James Watt School of Engineering, University of Glasgow, Glasgow, G12 8QQ UK; 2 Bangor College, Central South University of Forestry and Technology, Changsha, China

**Keywords:** IC recycling, PCB assembly, LCA, Sustainable electronics, Electrical and electronic engineering, Electronics, photonics and device physics

## Abstract

As consumer microelectronics become ever more ubiquitous, there are growing concerns about their environmental impact. However, the diversity of designs and components used in modern devices makes a coherent mitigation strategy hard to formulate. In this work, we perform a quantitative life cycle assessment (LCA) of the environmental profiles of both high-value (a smartwatch) and low-value (a TV remote) devices and find that the optimal mitigation strategy varies substantially between these two extremes. We find that the impact of the smartwatch is dominated by the production costs of its integrated circuits (ICs), and so a priority on device lifetime and design-for-recycling of the ICs is the best path to minimizing impact. On the other hand, the TV remote’s impact is dominated by the cost of its fiberglass (FR4) substrate, with the much simpler ICs playing a much smaller role. Our results show that the impact of low-cost devices is best mitigated by incorporating eco-friendly substrates and additive manufacturing techniques, while also minimizing the use of critical raw materials (CRMs). These results will help guide future industrial strategies, and we provide a list of challenges and opportunities in making electronics green.

## Introduction

In the past 50 years, there has been remarkable progress in electronics which has sparked a revolution across various sectors, including communication, agriculture, aerospace, manufacturing, and consumer technology^1,2^. Consequently, there is an ever-increasing demand for electronics, leading to a 50% increase in the usage of Integrated Circuits (ICs) in the last five years within consumer devices; a trend that is predicted to continue growing at a similar rate^1^. This is driven by increasing demand as well as the increasing complexity of electronic devices. Most consumer electronics possess the basic building blocks of CPU, RAM, and long-term memory, but also include ICs for an increasing number of other functions, such as sensing, actuation, and power management. For example, the list of sensors considered essential within a smartphone is only increasing^3^. However, a problem remains that the circular economy for electronics remains relatively immature compared to other industries; manufacturing, integration, usage and End-of-Life (EoL) processes adhere to a wasteful, linear and inefficient economic model^4^.

Therefore, most ICs end up in landfills^5^ after an average useful life of less than five years^6^. Within many products, devices are rarely discarded due to IC failure or obsolescence; this is much more likely due to damaged screens, battery degradation or consumer preference to upgrade, amongst other factors^7^. In addition to the ICs, a large portion of WEEE (∼42% by weight) is also comprised of printed circuit boards (PCB)^4^, which typically use a substrate such as Flame Retardant 4 (FR4)—a composite material consisting of woven fiberglass cloth with an epoxy resin binder. FR4 stands as a highly utilized substrate material in PCBs owing to its electrical and mechanical characteristics but accounts for a substantial portion (∼70%) of waste PCBs by weight. The FR4 substrate presents a significant environmental barrier for the circular economy due to its composition of toxic elements like brominated flame retardants (BFRs) and non-metallic materials (such as glass fiber and epoxy resin)^8^. As such, there is increasing R&D into alternative substrates such as paper, biodegradable polymers and wood^9^, and these have been adopted in various IoT and wireless research demonstrators^10^. These materials are tested for their compatibility with existing processing techniques as well as their potential for additive manufacturing and degradability. Substrate materials have been developed explicitly for electronics, such as the Soluboard^®^ by Jiva technologies, which is based on naturally derived materials that can be dissolved in hot water to recover high-value metals and discrete components^11^. Such solutions enable a “design for recycling” approach that would enable the easy recovery and recycling of materials and components such as ICs from WEEE items. However, quantitative Life Cycle Assessments (LCAs) are required to assess their true impact and cost/benefit ratios. Thus far, most electronics LCA focus on the use of FR-4 based electronics, cables^12^ or full systems^13^, with only limited studies on the impacts of Designing for Recycling and Reuse of components. Recently, there have been an increasing emergence of reports on the LCA of semiconductor devices across processes^14^, including the application of these LCAs to new wireless sensing systems^15^. Integrating discussions on IC recycling within the broader scope of LCA studies has been done. By focusing on the recycling of ICs, we aim to demonstrate effective strategies to reduce direct environmental impacts and enhance sustainability in electronics manufacturing.

In this paper, we first present a quantitative life cycle assessment (LCA) of the environmental impacts associated with different consumer electronics, specifically focusing on a high-end electronic device—a smartwatch—and a relatively simple device—a TV remote. These devices were selected because they represent a range of complexities in consumer electronics, allowing for a focused analysis on the impacts of IC recycling and substrate choice without the confounding effects of components like large glass panels found in more substantial devices. The focus of this work is on the Waste PCB, which is estimated to take around 42% of the weight of WEEE^4^, and also pose a major environmental threat due to the inclusions of toxic substances, such as brominated flame retardants (BFRs). Our focus is how we transition to a more sustainable PCB. Each case study investigates the impact of substrate choices and the benefits of recycling components on their overall environmental profile. Subsequently, we detail our methodology, including the LCA model specifics and environmental impact categories assessed. Results are then discussed, illustrating the environmental implications of substrate selection and IC recycling for both case studies. Finally, we discuss broader industry implications, including challenges and opportunities for enhancing sustainability through IC recycling and substrate innovation.

## Methodology: LCA analysis

This study compares the environmental impact across the life cycle of two consumer electronic products, the smartwatch and a TV remote control, to investigate how to improve the environmental profile of both products and, from these findings, propose changes that will reduce the environmental impact. The scope of this research will cover an initial model based on current PCB manufacturing processes and materials and a revised model with a lower environmental impact. The study will focus on analyzing materials and the processes needed to enhance the sustainability of PCB manufacturing. Models were created in a commercial software package “GaBi” from Sphera, Germany. GaBi models are derived from the inputs and outputs of each phase of the manufacture. Our LCA model also quantifies energy and water consumption, critical in PCB manufacturing processes such as etching and cleaning. We integrate specific data on these resources to ensure an accurate representation of their environmental impacts, considering variations in regional energy mixes and water treatment practices.

The environmental impact assessments were based on the Centrum voor Milieuwetenschappen Leiden (CML) impact categories and cover the Acidification potential (AP), Abiotic depletion potential (ADP), Eutrophication potential (EP), Freshwater Aquatic Ecotoxicity Potential (FAETP), Global warming potential (GWP), Human Toxicity Potential (HTP), Marine Aquatic Ecotoxicity Potential (MAETP), Ozone depletion potential (ODP), Photochemical Ozone Creation Potential (POCP) and Terrestrial ecotoxicity Potential (TETP) using the EcoInvent database. Global differences in energy mix and landfill procedure will impact the results. This study will constrain the PCB life cycle within Great Britain (GB) as keeping manufacturing local minimizes transportation emissions. Therefore, the work has applied the GB energy mix and utilized GB and European standard procedures for landfills.

Our initial modeling of FR-4 is based on previous studies from Babuna et al.^16,17^. Custom-made processes were built to accommodate the diversity of the manufacturing stages. These processes consisted of manufacturing an FR-4-based PCB and additive manufacturing processes. To assess the impact of ‘recyclable’ substrates, the only significant change was the substrate (i.e. not components, solder or process energy).

## Results

### Smart watch case study

Smartwatches were selected as a case study based on the rationale that it is a rapidly growing market, and use multiple ICs. The global smartwatch market was valued at USD 42.7 billion in 2022, with an anticipated compound annual growth rate (CAGR) of 14.5% from 2023 to 2032^18^. This growth suggests that the market value of smartwatches may approach that of smartphones in the next decade. Therefore, it is crucial to understand the environmental implications of their life cycle, including recycling. The impact of substrate selection is also considered to reduce the environmental profile of these two case studies.

#### LCA bill of material (BOM)

**Fig. 1:**
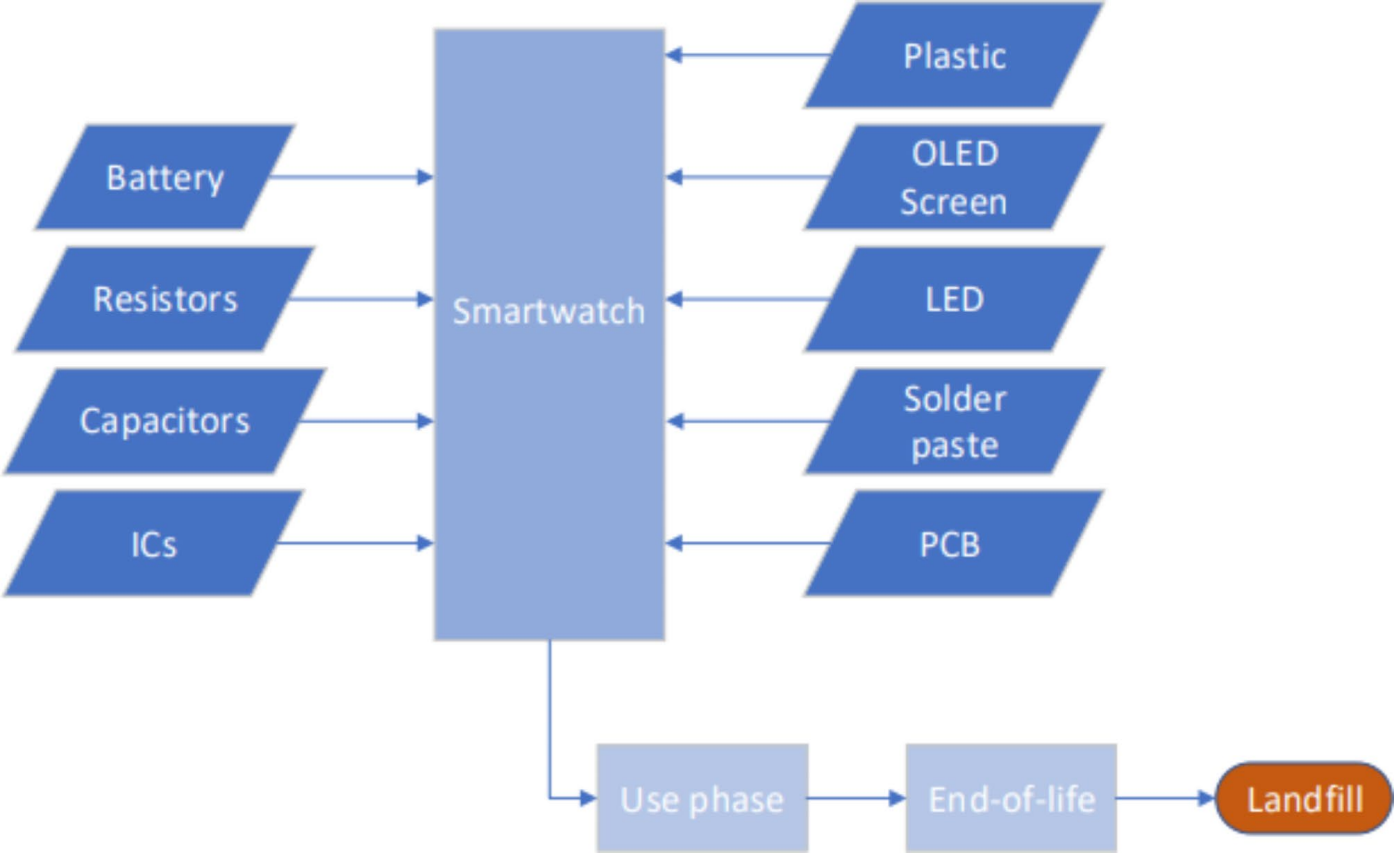
Life-cycle assessment flow diagram for the smartwatch without any recycling.

The inventory of components was constructed from a teardown analysis of a Google smartwatch conducted by TechInsights^19^. The inventory was estimated by processes available in Sphera’s GaBi software, shown in Table 1. All ICs are as close as possible, with options chosen based on wafer size, process node, and function. Some of the GaBi components are scaled to match the approximate wafer size of the real component. This inventory acts as a representation of a smartwatch. In the control version, all parts of the smartwatch go to landfill - shown in Fig. 1 - and in the recycling scenario, all ICs are recycled for reuse in another watch at a rate of 90%. The recycling scenario includes the energy required to desolder the ICs from the PCB, which is assumed to be equal to the input soldering electricity.

**Table 1:** Input inventory for smartwatch life-cycle assessment.

Component	Quantity	Details
Printed Wiring Board 2-layer	7.07 cm^2^	Standard PCB
Capacitor ceramic MLCC 0603	80	Representative for all capacitors
Resistor flat chip 0402	462	Representative for all resistors
Solder paste SnAg3Cu0.5	3 g	
OLED screen	7.3 cm^2^	Self-made (see supplementary)
Cell BR series (Li/poly-carbon monofluoride)	6 g	Battery substitute
LED SMD high-efficiency with lens	1	
Plastic extrusion profile	18 g	Case and strap substitute
IC BGA 48 (72 mg) 8 × 6 mm MPU	1	Main processor substitute
Filter SAW (25 mg) 3 × 7 × 1	3	RxD frontend component
IC DFN 10 (22.3 mg) 3 × 3 mm CMOS	1	RxD frontend component
Transistor signal SOT-883	1	Infineon switch substitute
IC BGA 48 (70 mg) 6 × 6 × 1.1 mm	1	Power amplifier substitute
IC WLP CSP 49 (10.2 mg) MPU generic	1	RF chip substitute
IC BGA 144 (181 mg) 10 × 10 mm	0.01	Power tracker substitute
IC WLP CSP 49 (10.2 mg) CMOS logic	8	Co-processor substitute
Soldering electricity	0.533 kWh	
Use-phase electricity	3.86 kWh	Assumes battery fully recharged every day for 3 years

#### LCA results

**Fig. 2:**
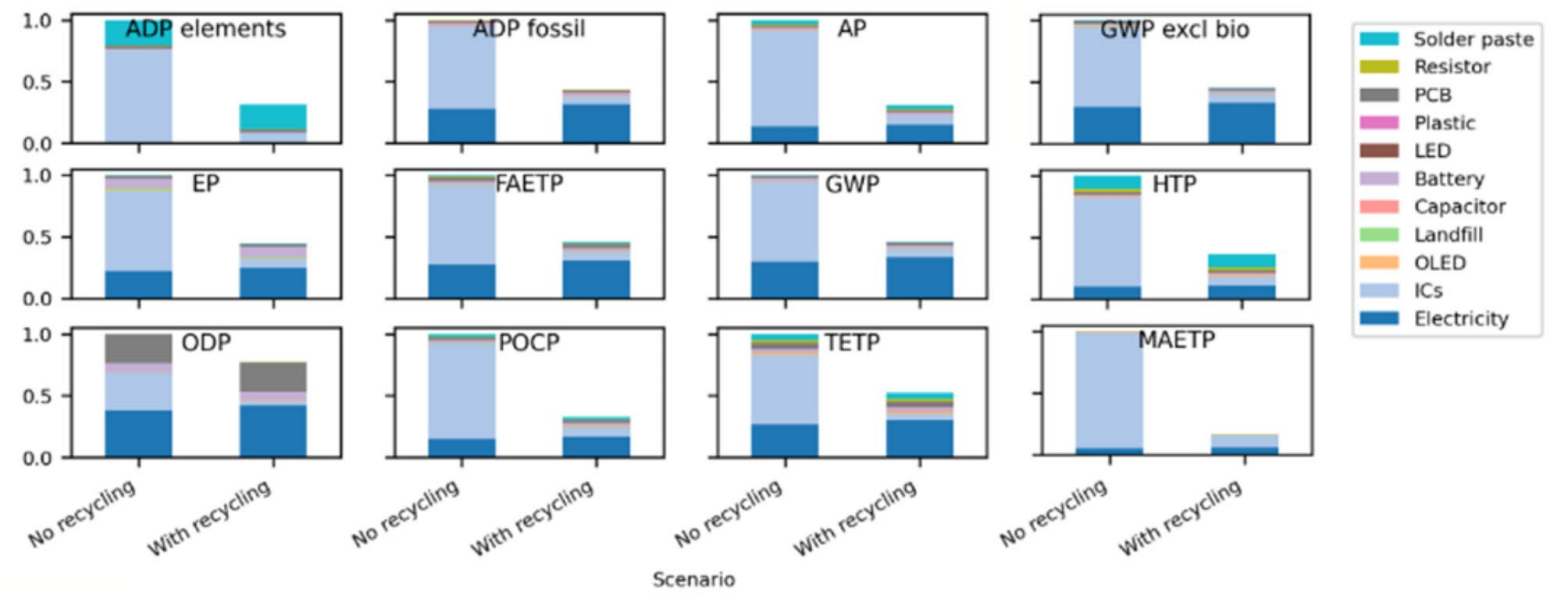
Comparative LCA results for all CML impact categories. In all plots, the left bar shows the normalized value without recycling, and the right bar shows the value with recycling included. The bars are scaled as a proportion of the maximum value in each plot.

The environmental impacts were calculated by the software using the CML methodology, and the results are shown in Fig. 2. In all impact categories, the ICs are shown to be the dominant source within that impact category. The source of this is discussed in multiple studies, e.g^14^. but is due to IC manufacture being highly input-intensive Clearly, decarbonization of semiconductor processing is needed. However, Scenario 2 has considered the impact of IC reuse on the environmental profile by the adoption of a PCB similar to the Soluboard^11^ (i.e. the “Recyclable PCB”). To do this, the Waste PCB needs to be placed in boiling water, and the IC requires solder removal and reattachment onto a new substrate, all of which were set up as additional manufacturing processes in the LCA model.

As expected, the environmental impact is lower in all categories for the scenario which uses the recyclable board with reused components (see Fig. 2). The benefit of using this is seen, for example, in the GWP category. As the ICs are clearly the largest contributor to the environmental impact, the adoption of a recyclable substrate enhances the ability to recover and reuse the ICs, dramatically lowering the overall impact by over 50%. Similar reductions are seen in all impact categories, with the greatest being MAETP, which shows an 84% reduction due to the reduction in process chemicals needed for component manufacture.

**Fig. 3:**
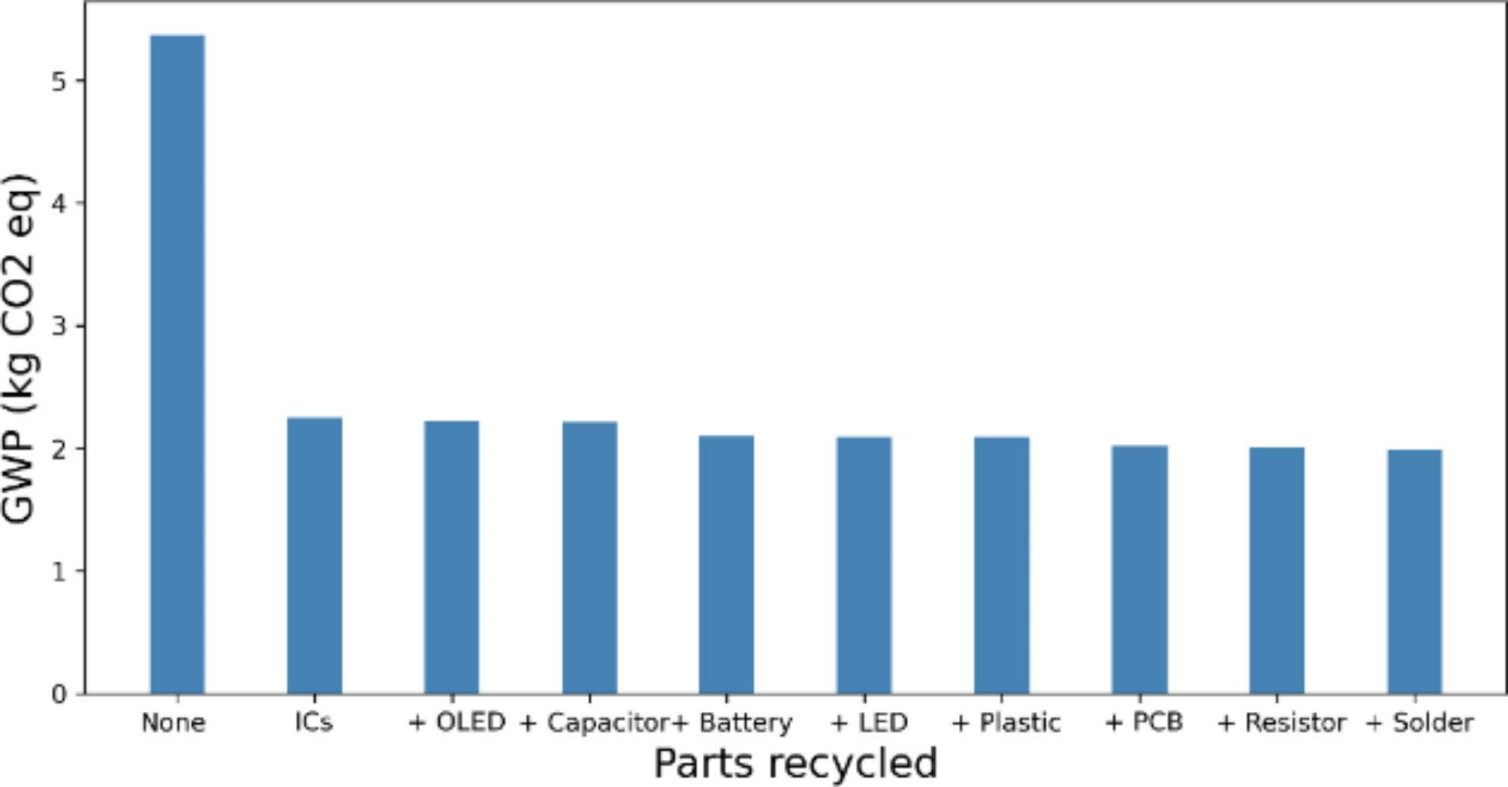
GWP of the partially-recycled smartwatch showing the significance of IC recycling, compared to other components when reducing the smart watch’s overall GWP.

To show the specific impact of recycling different components, the GWP is plotted as a function of the component in Fig. 3. The recycling of the ICs has the largest single impact on the GWP of the device, with all other components having a marginal impact. Nevertheless, recycling all components reduces the overall GWP impact of the smartwatch by 63%, compared to 56% by just recycling the ICs.

In the case of the smartwatch, the substrate itself has a negligible effect on the total LCA impact due to its small footprint. Because of this, no other substrates (e.g. paper or PET) were investigated because they would add significant manufacturing complexity while not improving the LCA results with any significance.

### TV remote PCB case study

#### TV remote BOM

The second case study was a TV remote control which is intended to show the environmental impacts of simpler electronic circuits found in consumer electronics. The components and dimensions are based on the teardown of a TV remote^20^. Some TV remotes, e.g. Amazon Fire TV remote, are more complex and contain a higher number of IC components^21^ due to Bluetooth functionality. However, for this case study, the function of the remote is limited to infrared (IR) signaling to the TV, and the inventory is shown in Table 2. In comparison to the smartwatch case study, the PCB substrate represents a much larger proportion of weight in comparison to the discrete components used in the LCA model. As such, it is of greater interest to explore the environmental impacts of the different PCB substrates, as well as the recycling of the IC.

LCA plans for four different substrates have been created. The control is a standard FR4 substrate, and the alternative substrates are based on paper, Polyethylene Terephthalate (PET) and a recyclable substrate; similar to the principle of the Soluboard by the Jiva Technologies examples. In total, four scenarios are investigated.

- Scenario 1; using standard PCB material of FR4 is used, along with SnAgCu solder. The entire PCB is assumed to end up in a landfill after the use phase; referred to as ‘FR4’.
- Scenario 2; using a PET substrate and swapping the subtractive copper with printed silver conductive ink for conductive tracks and using a silver conductive adhesive as the die-attached material. The end-of-life is also assumed to be landfill; referred to as ‘PET’.
- Scenario 3; using a Paper substrate and also uses printed silver for conductive tracks and attach material The end-of-life is also assumed to be landfill; referred to as ‘Paper’.
- Scenario 4; using the degradable substrate material, which degrades at EoL in hot water allowing for the recovery of 90% of the ICs (assuming a small proportion are non-recoverable); referred to as ‘Degradable.’

**Table 2:** Input inventory for tv remote PCB life-cycle assessment - control scenario.

Component	Quantity	Details
Printed Wiring Board 1-layer rigid FR4	40 cm^2^	Standard PCB
Solder paste SnAg3Cu0.5	66 mg	
LED SMD high-efficiency with lens	1	IR LED substitute
IC SSOP 14 (120 mg)	1	
Resistor thick film flat chip 0402	3	
Transistor signal SOT23 3 leads	1	
Diode power DO214/219	1	
Capacitor ceramic MLCC 1210	2	
Soldering electricity	0.115 kWh	

#### LCA results for TV remote PCB

**Fig. 4:**
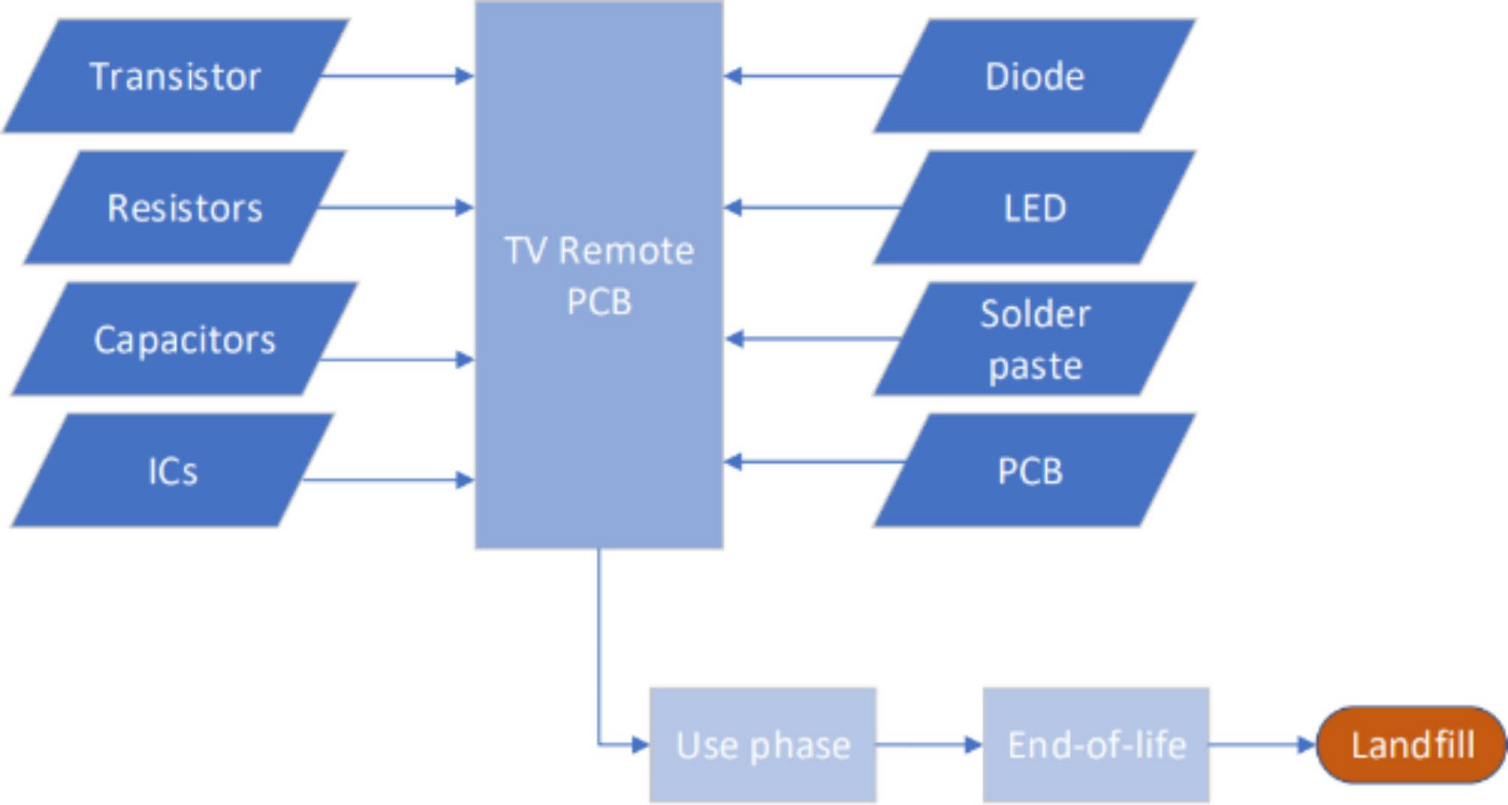
Life-cycle assessment flow diagram for the TV remote PCB without any recycling.

**Fig. 5:**
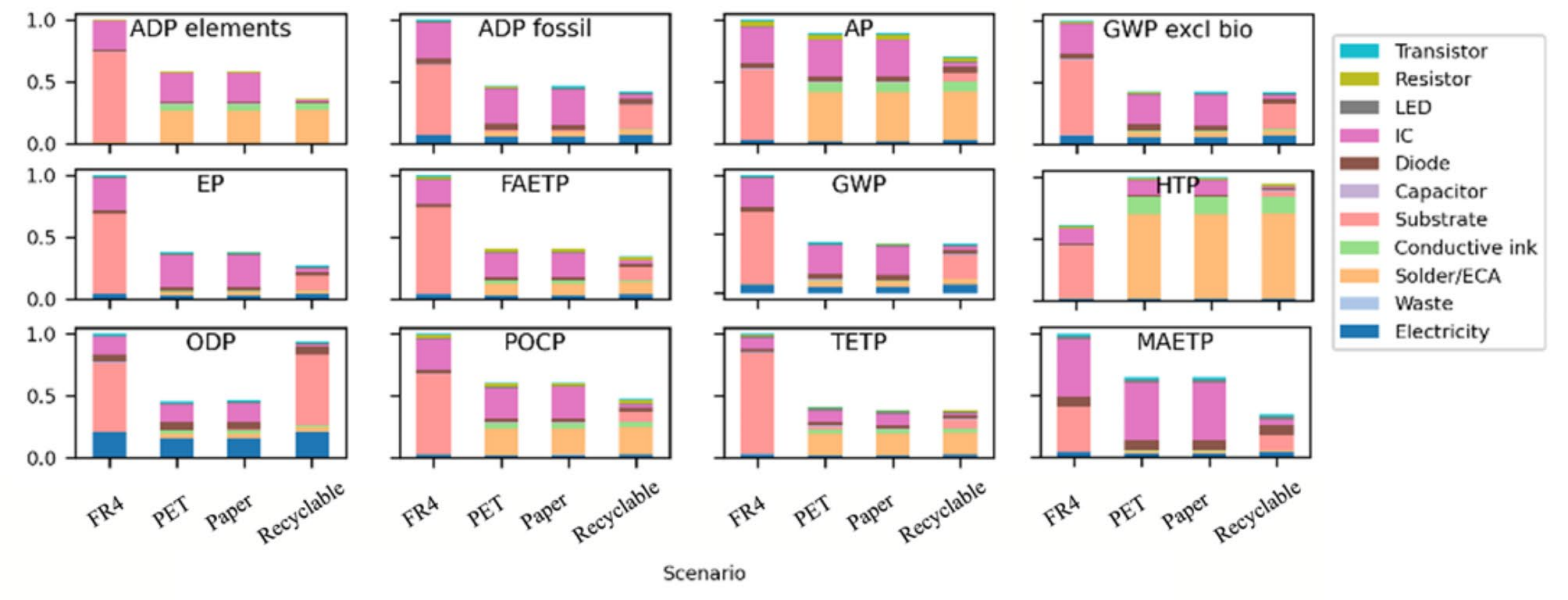
Comparative LCA results for all CML impact categories.

The environmental impacts of the four different scenarios were analyzed using the CML methodology and are based on the flow diagram in Fig. 4. The relative results for all impact categories and scenarios are plotted in Fig. 5. In each plot, the scenarios represented by each bar are in the order: FR4, PET, paper, Degradable.

Considering the FR-4 LCA results, the largest contributing components are shown to be the substrate, followed by the IC (see Fig. 5). The FR4 scenario has the highest GWP impact from all four scenarios, due to the high substrate contribution as well as lack of component recycling, as this scenario assumes components are sent to landfill By contrast, the paper and PET LCA results are shown to be lower in most categories than the FR-4 scenario, except for HTP impacts, which is a result of the use of printed silver. The HTP impact category is higher because the production of silver is a high-consumption process and this impact increases due to SO2 emission from fossil fuel combustion as well as the cyanide, biphenyl, mercury, lead, and tin discharged during the refining process. Overall, the printed (‘additive’) manufacturing process used in the paper- and PET-based PCBs and the lower impact of the substrate material ensures PET or paper-based electronics have a lower impact than FR4 in most other impact categories. The results highlight the importance of substrate material for electronic products where a relatively large PCB footprint is presented, such as in this case study.

The final scenario (‘Degradable’) has the lowest GWP, but it is worth highlighting it only has a minor improvement in overall GWP of the degradable scenario and paper scenario. The higher substrate contribution from the degradable substrate is offset by the reduced IC contribution. However, it shows reduced ADP, AP, EP, FAETP, MAETP and POCP compared to the paper- and PET-based PCBs as it allows for rapid recovery and reuse of components.

### Discussion on the challenges of reducing IC’s environmental profile and of reuse

The results highlight the greatest impact of two consumer products; the ICs dominate the smartwatch environmental impact and changing the substrate has only a minor impact. By contrast, on a simpler consumer device such as a TV remote, the substrate has a much greater influence in most impact categories. Sustainable substrate selection is critical, but these are at the R&D stage and not in commercial usage; examples include using biodegradable and natural materials such as banana fabric epoxy composites, wood-based substrates, and innovative uses of agricultural byproducts^9,22–26^. In any case, as we transition to more complex and compact consumer electronic devices, it is clear that the ICs are going to remain as the most significant hotspot within many products. Reuse is always preferable to recycling/material recovery in the waste hierarchy, and the LCA data shows how significantly reused ICs can improve the environmental impact of electronic products. For instance, it has been noted significant environmental impacts from optimizing material usage in smart labels^27^ and conductive inks^28^ respectively, which aligns with our findings regarding the criticality of material choices. Therefore, this section focuses on how we can use the main conclusions of the data from Sections “Results” and “Conclusion” to reduce the environmental profile of electronic products through IC recycling by considering the technical and economic challenges ahead. Additionally, studies such as Glogic et al.^29^, which demonstrated the environmental benefits of using recyclable substrates in electronics, support the critical importance of sustainable material selection.

#### IC recycling process

Component recovery in electronics is often not an integrated facet of many recycling operations due to the associated costs, the complexity of the processes, the fundamental nature of conventional recycling practices, and uncertainties regarding the performance of reclaimed materials. In order to introduce this into a recycling facility, a pre-processing stage needs to be introduced, as shown in Fig. 6, where the components are removed from the surface of the Waste Printed Circuit Boards (WPCBs) using a two-step approach. The first is the removal of the solder to release the component from the substrate. The second step is use of an external force to separate the substrate and the released components. IC recycling is currently undertaken only for high-value or specialist electronics applications such as military or space, or where new components are obsolete^30^. Other examples of IC recycling tend to occur downstream of developing nation recycling facilities. Clearly, the decision to undertake IC recycling is currently driven by financial reasons; however, this could significantly decarbonize electronics products. The steps to recycle ICs is dissected into three critical stages:

1. Desoldering process
  For desoldering ICs from WPCBs, there are three main options; removal via (1) mechanical grinding of solder joints on the rear of the PCB; (2) removal via chemical separation; or (3) thermal removal of the solder via infrared heaters, hot air, supercritical fluid methods or hot liquids^31^. Of the three approaches, mechanical grinding is not suitable for Surface Mount Technology (SMT), which now dominates over Through Hole Technology (THT)^32^. Solders contain varied material mixes, which often makes chemical removal of the solder sometimes difficult; especially without inadvertently removing other metals on the PCB such as surface coatings, copper tracks, etc. However, when the chemistry of the solder is known, SML can offer a selective separation between metal and nonmetals of WPCBs.
  The thermal removal method provides the greatest benefit in terms of environmental impact, efficiency and cost^31^. Difficulties occur as WPCBs are highly heterogeneous and complex and therefore, differences in heat capacity and inhomogeneous distribution between components can lead to an inconsistent temperature increase in different regions of WPCBs during heating. This can destroy the ICs during desoldering. Heat can also cause moisture diffusion and expansion inside the chip, resulting in stresses greater than the internal interlayer bonding force, thus causing delamination defects in the chip^33^.
2. Disassembly process
  The disassembly process requires mechanical removal, which is typically done by vacuum suction, high voltage, shaking/vibration, gas jet removal, or the use of centrifugal or shear force. Clearly, the PCB structure, whether SMT or THT impacts the disassembly process selection. Vertical force is most effective when removing devices made with THT, while a horizontal (‘shear’) force was more effective when removing surface-mounted devices (SMDs)^31^. High voltage separation in PCB disassembly leverages electrical potential to isolate components after solder melting, offering a non-contact approach that minimizes thermal stress and potential damage to the PCB and components^32^, though further tests are needed on degradation after subjecting to high Voltages.
3. Component reworking
  For most SMDs, it’s crucial to remove residual solder for a clean component-board interface, which is essential for solder wetting and strong bond formation via an Inter Metallic Layer (IML). The IML, usually under 25 μm in thickness, is critical for bond strength and there is a limited understanding of how residues from previous solder joints affects the bond strength and joint reliability in new joints^34^.
  **Fig. 6:**
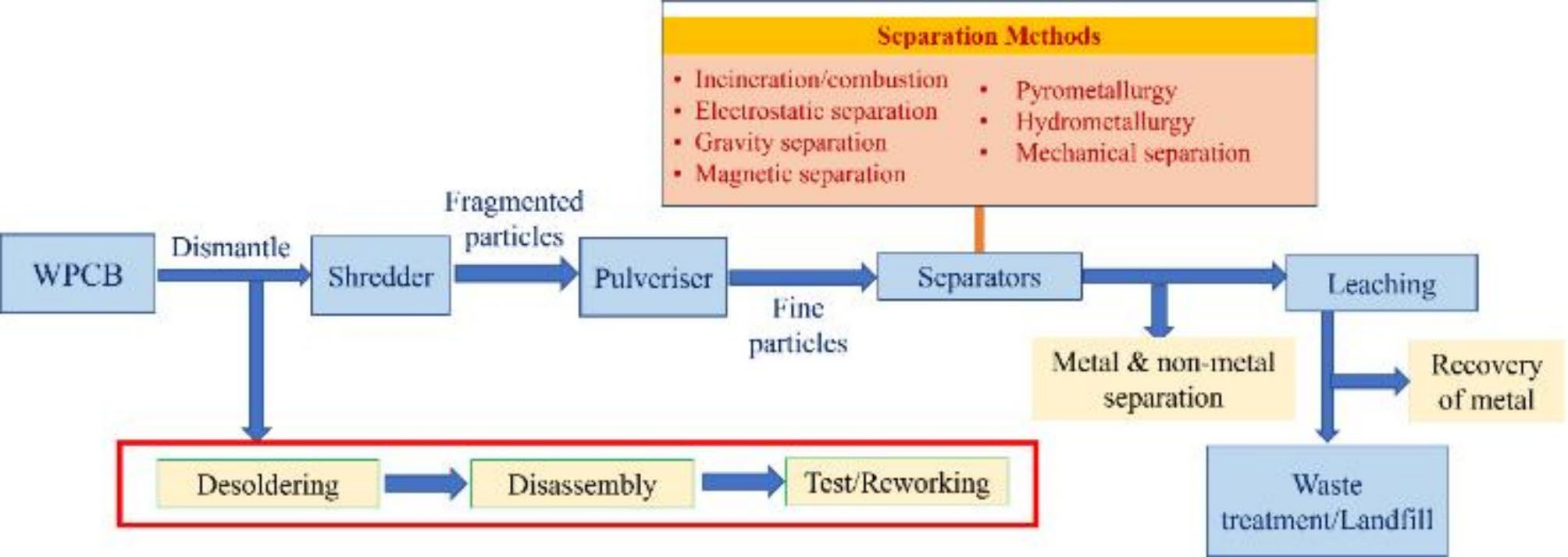
Recycling process highlighting the steps required for a complete recycling process involving component recycling.

IC re-balling is also required in the case of BGA packages and involves applying new solder to component pads to ensure a strong IC-PCB connection post-repair or modification^35^. Upon re-balling BGA packages, it’s critical to validate the rework by testing and inspecting the ICs to ensure their reliability and functionality^36^. This requires electrical testing and the use of Non-Destructive testing (NDT) approaches, such as X-ray imaging, to detect potential soldering defects that could affect performance^37^. While these steps are established, there’s limited research discussing the bond strength outcomes when utilizing recycled components or materials in the re-balling process, presenting an opportunity for further study^38^.

### Opportunities in IC recycling

*Resource shortage/Scarcity*: The persistent global shortage of electronic components has caused considerable disruptions during and after the Covid pandemic. Companies can mitigate these shortages by reusing valuable components, reducing their dependence on new supplies and alleviating supply chain constraints.

*National security/Safeguard supply chains*: By reusing electronic parts, firms can mitigate the risks associated with volatile component availability and prices, maintaining consistent supplies and reducing production interruptions.

*Reducing E-waste*: Repurposing electronic components, such as integrated circuits (ICs), is vital for reducing e-waste and its environmental impact. Despite ICs making up a small fraction of e-waste, they are significant for their valuable metal content, notably gold, highlighting the importance of their recovery in minimizing resource loss^39^.

*New industrial opportunities*: The need for an “Electronics Component Testing Industry” could stimulate economic growth by generating new employment opportunities and driving the demand for electronic testing equipment through WEEE procurement and component resale. Companies are adopting Sustainable Business Model Innovation (SBMI) to foster practices that positively impact the environment and society, in line with Sustainable Development Goals (SDGs). In response to environmental concerns, new substrate materials are being explored to replace traditional FR4. However, it is not clear these alternatives match FR4’s durability, so this needs vigorous and assessment whether the lower lifetimes contribute to higher environmental profiles, despite having a lower impact at the manufacturing stage.

*Consumer behavior change*: Consumers may offset some of their expenses for electronic devices by selling WEEE, improving formal recycling systems and incorporating informal recycling networks, which could benefit the overall economy and reduce the occupational hazards associated with these jobs.

*New Modular electronics and life extension methodologies*: The development of new modular electronics approaches to remove and reuse ICs could enhance product longevity and potentially create new employment avenues within the electronics sector.

*LCA*: As indicated in Section “Results”, reusing components is a proactive approach to creating environmentally friendlier electronic products, reducing carbon output, and redefining manufacturing for the low-carbon sector.

### Negatives/Risks in IC recycling

It is technically feasible to recycle ICs, and the environmental benefits are quantified in Sections “Results” and “Conclusion”. This section highlights the opportunities and threats that IC recycling poses to the semiconductor industry.

Recycling ICs, while beneficial for the environment and resource conservation, carries several risks and challenges for consumers and the industry.

*Proprietary information on ICs*: The reluctance to share proprietary IC data due to intellectual property rights can pose significant challenges to reprogramming or reconfiguration.

*Skills and automation requirements*: Automation technology is needed due to the vast scale and to prioritize high-value ICs. Training of staff in recovery, reworking and testing are necessary.

*Assessing remaining useful life*: The task of accurately estimating the remaining useful life (RUL) of recycled integrated circuits is compounded by the absence of historical data, casting doubts on their reliability. This uncertainty could potentially undermine confidence in the viability of these components for future applications.

*Desoldering failures*: Desoldering, a critical step in the recycling process, poses a significant risk of thermal damage to ICs, potentially compromising their integrity and functionality.

*Cost challenges*: The economic rationale for recycling ICs, particularly those of lower value, is a challenge. The overheads related to collection, sorting, and processing can often outweigh the financial benefits derived from the materials reclaimed, challenging the sustainability of large-scale recycling without innovative cost-reduction strategies. Moreover, as suggested in the paper^40^, the utilization of biodegradable materials such as PLA in electronics could potentially lower production and recycling costs by simplifying the manufacturing process and reducing the need for complex end-of-life material separation.

*Need for quality assurance*: Ensuring the quality of recycled ICs requires a comprehensive framework of strict quality assurance measures. This includes detailed inspections, advanced automated testing, and thorough safety evaluations, exemplified by practices such as Destructive Physical Analysis (DPA). These measures are critical to affirm the components’ compliance with functional and safety standards, thereby sustaining trust among end users.

*Potential for fraud*: There is a risk of fraud in IC recycling, with refurbished ICs potentially being falsely marketed as new. This is a particularly acute concern in the high-value processors and memory chips market. Addressing this challenge necessitates the implementation of robust verification mechanisms and the maintenance of transparency throughout the recycling supply chain, ensuring the integrity of the process and the authenticity of the recycled components.

*National security and end-use*: Repurposing ICs for military uses raises security issues, particularly where sanctions exist against using Western technology in foreign military hardware. Innovative tracking and regulation of IC end-use are crucial to prevent misuse and align with global security protocols.

## Conclusion

The environmental impact of IC manufacturing is notable, given the industry’s reliance on rare metals and energy-intensive processes. This paper shows the results of a detailed LCA study into the impact of this and the substrate selection. A comparative scrutiny of two electronic devices, including a smartwatch and a TV remote, reveals the distinct environmental footprints of ICs versus other components. In the case of the smartwatch, the IC chip manufacturing accounts for the largest share of the impact, dominating over the product use phase and the substrate. By contrast, the results from the TV remote showed the substrate to have the largest impact. In both cases, IC recycling was shown to significantly reduce the overall product footprint by > 50%.

Such analyses advocate for an industry-wide pivot towards embracing recyclable components ensuring better sustainability of electronic products. Clearly, there are challenges to adopting reused components in electronics manufacture; nevertheless, this paper highlights that this could provide substantial reductions in the industries manufacturing footprint.

## Data Availability

The datasets used and/or analysed during the current study available from the corresponding author on reasonable request.
